# Mapping of QTL for total spikelet number per spike on chromosome 2D in wheat using a high-density genetic map

**DOI:** 10.1590/1678-4685-GMB-2018-0122

**Published:** 2019-11-14

**Authors:** Mei Deng, Fangkun Wu, Wanlin Zhou, Jing Li, Haoran Shi, Zhiqiang Wang, Yu Lin, Xilan Yang, Yuming Wei, Youliang Zheng, Yaxi Liu

**Affiliations:** 1 State Key Laboratory of Crop Gene Exploration and Utilization in Southwest China, Wenjiang, Chengdu 611130, China.; 2 Triticeae Research Institute, Sichuan Agricultural University, Wenjiang, Chengdu 611130, China.

**Keywords:** recombinant inbred line, synthetic hexaploid wheat, quantitative trait locus, total spikelet number per spike

## Abstract

Total spikelet number per spike (TSS) is one of the key components of grain yield in wheat. Chromosome (chr.) 2D contains numerous genes that control TSS. In this study, we evaluated 138 F_8_ recombinant inbred lines (RILs) derived from an F_2_ population of a synthetic hexaploid wheat line (SHW-L1) and a common wheat cultivar (Chuanmai 32) for TSS in six different environments. To identify quantitative trait loci (QTL) for TSS, we constructed an integrated high-density genetic map of chr. 2D containing two simple sequence repeats, 35 diversity array technology markers, and 143 single nucleotide polymorphisms. We identified three stable QTL for TSS that individually explained 9.7–19.2% of the phenotypic variation and predicted 23 putative candidate genes within the QTL mapping interval. Overall, our results provide insight into the genetic basis of TSS in synthetic hexaploid wheat that may be useful in breeding high-yielding wheat cultivars.

## Introduction

To feed the ever-growing population, improving the yield of wheat, one of the most important food crops globally, is becoming increasingly important (Rajaram, 2001; Godfray *et al.*, 2010; Reynolds *et al.*, 1996). Among the factors determining wheat yield, total spikelet number per spike (TSS) is considered one of the key factors, and previous studies have shown that spikelet number is closely related to grain number (Rawson, 1970) and determines where spikelets can set (Slafer and Andrade, 1993). As the basal units of inflorescences, spikelets are crucial for reproductive success and final yield (Cai *et al.*, 2014).

TSS, as an important quantitative agronomic trait, is controlled by polygenes and influenced by the environment (Zhou *et al.*, 2017). Understanding the genetic factors underlying variations in TSS without environmental interference is essential for the genetic improvement of wheat (Mackay, 2001; Wurschum, 2012). Previous genetic studies have revealed that chromosome (chr.) 2D is rich in genes that control spikelet number per spike in common wheat, and many quantitative trait loci (QTL), such as *QSsn.cau-2D.2* and *QSpn.nau-2D*, have been discovered on this chromosome (Ma *et al.*, 2007; Zhai *et al.*, 2016). However, information about TSS-QTL on chr. 2D is still limited for synthetic hexaploid wheat (SHW), which contains a combination of genes from *Aegilops tauschii* and common wheat (*Triticum aestivum*), as well as novel functional genes (Mares and Mrva, 2008). Yu *et al.* (2014) identified a stable QTL (in the region wPt-6133-gpw4473) for TSS on chr. 2D in a population developed from a cross between SHW (SHW-L1) and the common wheat variety Chuanmai 32, using a genetic map containing simple sequence repeats (SSRs) and diversity arrays technology (DArT) markers. To accurately parse this QTL, we integrated the markers reported by Yu *et al.* (2014) with novel SNP markers into a new chr. 2D high-density genetic map and identified QTL for TSS. Our data might help to better understand the genetic basis of TSS in SHW and accelerate the development of new high-yielding wheat cultivars.[Bibr B39] [Bibr B40] [Bibr B41]

## Materials and Methods

### Plant material

A total of 138 F_8_ recombinant inbred lines (RILs) derived from an F_2_ SHW-L1/Chuanmai 32 population were used to construct an integrated linkage map for chr. 2D and detect QTL for TSS. SHW-L1 is an SHW derived from a cross between *T. turgidum* ssp. *turgidum AS2255* (AABB) and *A. tauschii* ssp. *tauschii AS60* (DD) (Zhang *et al.*, 2004), whereas Chuanmai 32 is a commercial hexaploid wheat cultivar grown in the southwest winter-wheat areas of China. Transgressing segregations for TSS have been previously observed in SHW-L1/Chuanmai RILs, and a total of 68 SSRs and 1794 DArT markers for important agronomic traits have been mapped (Yu *et al.*, 2014).

### Field experiment and phenotyping

All RILs and their parents were evaluated in a completely randomized block design with two replicates, at the experimental stations of Dujiang Weir (31°01’N and 103°32’W) in 2008, 2009, and 2010 (environments E1, E2, and E3, respectively), Guanghan (30°99’N and 104°25’W) in 2009 and 2010 (environments E4 and E5), and Wenjiang (30°36’N and 103°41’W) in 2011 (environment E6). Plants were sown in single 1.5-m rows with a 30-cm space between rows and a 10-cm space between individuals. Data for TSS were manually counted from 10 randomly selected guarded main spikes from each line in each environment (Yu *et al.*, 2014).

### Statistical analysis

To estimate random effects, a best linear unbiased prediction (BLUP) mixed model was used to obtain BLUP-TSS values (Piepho *et al.*, 2008). The BLUP for the phenotypic value of plant Y_i_ was calculated as follows: Y_i_ = X_i_ f+ a_i_ + e_i_, where f is a vector of fixed effects, X_i_ is an incidence vector, e_i_ is the environmental deviation, and a_i_ is the phenotypic value (Goddard, 1992). An analysis of variance (ANOVA) was performed using SAS 9.1.3 (SAS Institute, Cary, NC, USA) to estimate the effects of genotype on TSS. The estimated broad-sense heritability of TSS was calculated as follows: h = σ^2^ G/(σ^2^ G + σ^2^ e/r), where σ^2^ G is the genetic variance, σ^2^ e is the residual variance, and r is the number of replicates per genotype.

### Construction of a genetic map for chr. 2D

A total of 13 SSRs, 93 DArT markers, and 2306 SNPs reported in previous studies (Yu *et al.*, 2014; Yang, 2016) were used to construct a genetic map for chr. 2D. After the removal of redundant markers that were located on the same loci (Yang, 2016), the genetic map consisted of 13 SSRs, 86 DArT markers, and 244 SNPs. The remaining markers were assigned to linkage groups using Joinmap 4.0 (Van Ooijen, 2006) with a recombination frequency of 0.25–0.05. The final genetic distances were obtained using the Kosambi mapping function (Kosambi, 2016).

### QTL mapping

QTL screening was conducted using interval mapping (IM) in MapQTL 6.0 (Van Ooijen, 2009). Logarithm of odds (LOD) threshold values for IM were determined based on 1000 permutations to declare significant QTL at *p*<0.05, whereas QTL with LOD values <3.0 were excluded to ensure the authenticity and reliability of the reported QTL. QTL that explained more than 10% of variation in TSS were considered as major QTL.

### Prediction of candidate genes

To predict candidate/flanking genes, the interval flanking marker sequence was aligned via a BLAST search against the International Wheat Genome Sequencing Consortium and EnsemblPlants databases to determine the position with the highest identity and detect genes within the closed interval. To predict the function of the candidate genes, we conducted Gene Ontology (GO) annotation and Kyoto Encyclopedia of Genes and Genomes (KEGG) pathway enrichment analysis at *p*<0.05, using *Arabidopsis thaliana* as a background species, in KOBAS 3.0.

## Results

### TSS variation in RILs

**Figure 1:**
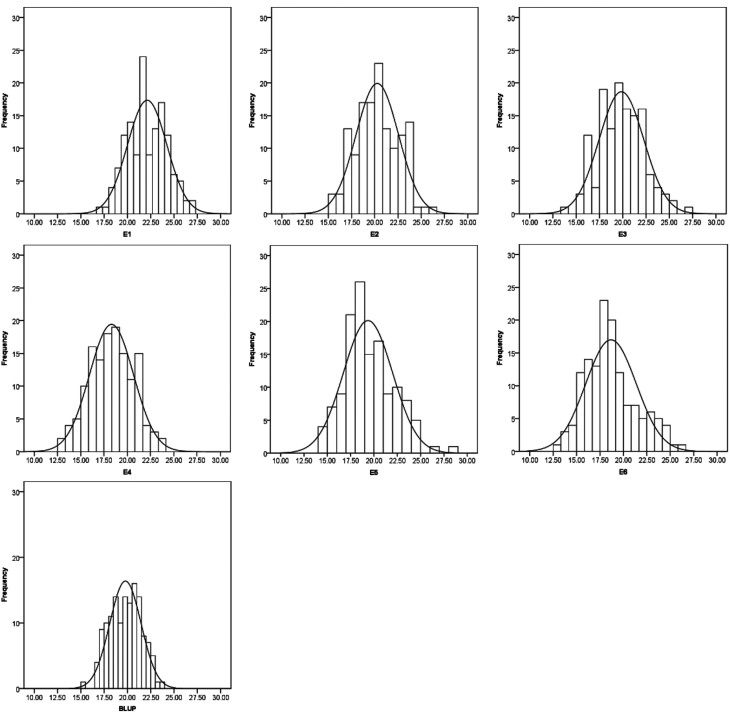
Frequency distribution of TSS in the SHW-L1/Chuanmai 32 recombinant inbred line (RIL) population under 6 environments. The horizontal axis indicates TSS value, the ordinate axis indicate frequency.

The results of the mean phenotypic performance and BLUP values for the TSS of RILs and their parents in the six environments are presented in Table 1. The ANOVA and heritability (h^2^) values are presented in Table 2. Variation among the RILs was high, with a coefficient of variation ranging from 9.53% in E1 to 14.39% in E6. Distributions were continuous across all environments (Figure 1), and, thus, the RILs were suitable for analyzing QTL for TSS.

**Table 1:** The mean phenotypic performance for TSS of the recombinant inbred lines (RILs) and their parents in six environments.

Environments	Parent		Population				
	SHW-L1	Chuanmai 32	Min	Max	Mean	SD	CV (%)
E1	-	-	16.80	27.20	22.12	2.11	9.53
E2	20.4	21	15.00	26.40	20.27	2.28	11.24
E3	21.6	20.5	13.60	27.20	19.86	2.40	12.07
E4	20.8	25	12.71	24.00	18.31	2.35	12.85
E5	21.2	22.4	14.00	28.00	19.36	2.63	13.57
E6	19.6	19.6	13.33	26.20	18.67	2.69	14.39
BLUP	20.9	21.8	15.49	23.85	19.78	1.67	8.46

**Table 2:** Analysis of variance (ANOVA) and the heritability (h2) values.

Degrees of freedom (DF)			Type III SS			Mean Square			F Value			Significance			Heritability (h^2^)
E	G	E*G	E	G	E*G	E	G	E*G	E	G	E*G	E	G	E*G	
2	139	278	785.34	2646.17	920.01	392.67	19.04	3.31	173.55	8.41	1.46	***	***	**	0.77

### Construction of genetic linkage map for chr. 2D

Different types of molecular markers were used to construct a genetic map for chr. 2D. At a maximum recombination frequency score of 0.4 and a minimum LOD score of 1.00, 180 markers were assigned to two different linkage groups (LG) that covered 207.33 cM, with a mean interval distance of 1.15 cM between the markers; however, the other 163 markers remained unassigned. LG 1 consisted of two SSRs, 35 DArT markers, and 90 SNPs, whereas LG 2 consisted of 53 SNPs.

### Stable QTL for TSS

**Figure 2:**
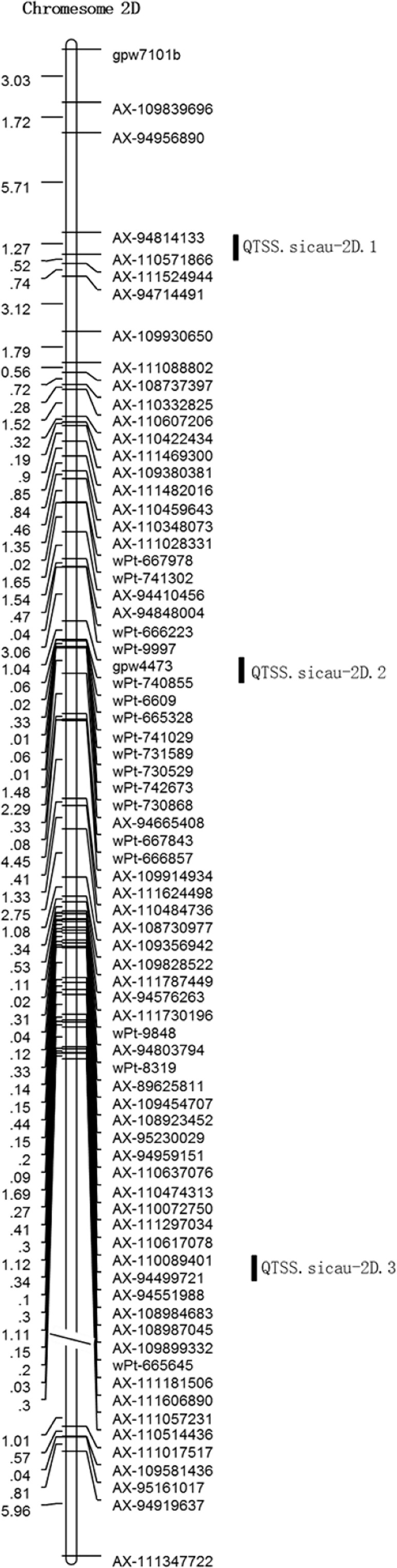
Chromosomal locations of quantitative trait loci for TSS and associated markers in the SHW-L1/Chuanmai 32 recombinant inbred line (RIL) population under 6 environments. The black bar points to the LOD peak of QTL.

Three QTL for TSS (*QTSS.sicau-2D.1*, *QTSS.sicau-2D.2*, and *QTSS.sicau-2D.3*) with significant effects in at least four environments were identified on chr. 2D. The existence and stability of the QTL were confirmed by BLUP values. The three major QTL individually explained 9.7–19.2% of the phenotypic variation (Table 3, Figure 2), and the additive effects of QTL showed that the positive alleles (i.e., those related to a high number of TSS) on chr. 2D originated from SHW-L1. Of these, *QTSS.sicau-2D.1* was detected in all environments, except E5, whereas *QTSS.sicau-2D.2* and *QTSS.sicau-2D.3* were found in four environments (E1–3 and E6).

**Table 3:** Quantitative trait loci (QTL) for TSS identified in the SHW-L1/Chuanmai 32 recombinant inbred line (RIL) population under 6 environments.

QTL	Environments	Marker interval	Nearest flanking marker	Max LOD	Combined LOD	% Expl.	Source
QTSS.sicau-2D.1	E1, E2, E3, E4, E6, BLUP	AX94814133-AX110571866	AX110571866	3.31-6.48	25.17	9.7-19.2	SHW-L1
QTSS.sicau-2D.2	E1, E2, E3, E6, BLUP	gpw4473-wPt740855	wPt740855	4.17-6.22	23.67	12.8-18.5	SHW-L1
QTSS.sicau-2D.3	E1, E2, E3, E6, BLUP	AX110089401-AX94499721	AX94499721	3.21-4.69	17.78	10.2-14.3	SHW-L1

### Putative candidate genes in QTL intervals

A total of 23 putative candidate genes associated with TSS were identified (Table S1). Two genes were predicted in the *QTSS.sicau-2D.1* interval (IWGSC_ref_V1_chr2D chr2D:9346330-9579108), 19 in the *QTSS.sicau-2D.2* interval (IWGSC_ref_V1_chr2D chr2D:38222754-43976070), and two in the *QTSS.sicau-2D.3* interval (IWGSC_ref_V1_chr2D chr2D:77381440-78089285). Of these, five genes, *LECRK42*, *AT2G34930*, *PME21*, *COBL7*, and *PIP5K4*, regulate flower development, and three, *CRK8*, *RPPL1*, and *AT4G29780*, are related to spikelet number differentiation. KEGG pathway enrichment analysis showed that *PIP5K4* is involved in inositol phosphate metabolism, phosphatidylinositol signaling system, and endocytosis; *PME21* and *PME53* are involved in starch and sucrose metabolism and pentose and glucoronate interconversions; and *AT2G07689* and *ATP1*are involved in oxidative phosphorylation.

## Discussion

**Figure 3:**
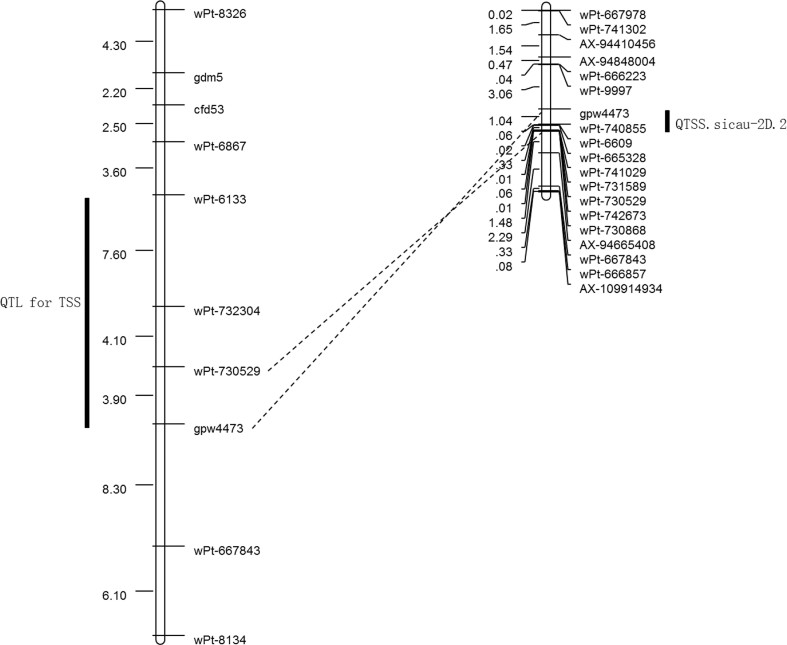
A comparison of stable putative QTL for TSS between a former study (Yu *et al.*, 2014) and our result. The left side shows the results of previous studies, and the right side shows the results of our studies.

In the present study, by using an integrated high-density genetic map, three major QTL for TSS were detected on chr. 2DS (short arm of chr. 2D). Among them, *QTSS.sicau-2D.2* was located in the marker interval *wPt6133*–*gpw4473*, which might correspond to that reported by Yu *et al.* (2014) in the same marker interval (Figure 3). Notably, using the integrated high-density genetic map for chr. 2D, we managed to decrease the marker interval range from 15.6 cM to 1.04 cM, which is a substantial improvement over that obtained in previous studies, and two additional QTL were detected. Similarly, by high-density consensus map, Marone *et al.* (2012) increased the map density from 11.8 cM per marker (as obtained by Nachit *et al.*, 2001) to 1.6 cM per marker, and Sourdille *et al.* (2003) confirmed previously detected QTL and identified three novel ones, suggesting that good coverage of chromosome is important for QTL detection. Therefore, this study provides a strategy for identifying QTL, which combines new molecular data with phenotypic data and enables possible detection of previously overlooked QTL.

For chr. 2D, previous studies have identified numerous putative QTL (Li *et al.*, 2002; Sourdille *et al.*, 2003; Quarrie *et al.*, 2006; Cui *et al.*, 2012; Liu *et al.*, 2014; Zhai *et al.*, 2016; Zhou *et al.*, 2017). Ma *et al.* (2007) reported two QTL for TSS in the marker intervals *Xwmc181.1*-*Xaf12d* (near IWGSC_ref_V1_chr2D chr2D:593738612-593738636) and *Xaf12*–*Xcfd239* (near IWGSC_ref_V1_chr2D chr2D: 647432804-647432824); Cui *et al.* (2012) also reported two QTL for TSS in the marker intervals *Xcfd267*–*Xmag3596* (near IWGSC_ref_V1_chr2D chr2D:608198901-608198921) and *Xbarc228*–*Xwmc181.1* (near IWGSC_ref_V1_chr2D chr2D:593738612-593738636). Zhou *et al.* (2017) also reported a QTL for TSS, nmed *QTsn.czm-2D.3* (near IWGSC_ref_V1_chr2D chr2D:480324893-480325330). Comparison with data in the IWGSC database revealed that these above-mentioned QTL were found on chr.2DL (long arm of chr.2D). The three QTL we detected were located on chr. 2DS; so, we paid more attention to the QTL previously detected on chr. 2DS. Li *et al.* (2002) reported a QTL for TSS in the marker interval *Xbcd611*–*Xgwm484* (IWGSC_ref_V1_chr2D chr2D: 34894502-48174395) on chr. 2DS; by comparison, this marker interval is different from those of *QTSS.sicau-2D.1* (IWGSC_ref_V1_chr2D chr2D:9346330-9579108) and *QTSS.sicau-2D.3* (IWGSC_ref_V1_chr2D chr2D:77381440-78089285), but contains *QTSS.sicau-2D.2* (IWGSC_ref_V1_chr2D chr2D:38222754-43976070). Zhou *et al.* (2017) also reported two QTL for TSS on chr. 2DS: *QTsn.czm-2D.2* was located in the marker interval *XPpd_D1*-*2DS_5382880_5243* (IWGSC_ref_V1_chr2D chr2D:29716047-67557838) that contains *QTSS.sicau-2D.2*, while the other QTL, named *QTsn.czm-2D.1* (IWGSC_ref_V1_chr2D chr2D:19623154-29716165), was different from *QTSS.sicau-2D.1* and *QTSS.sicau-2D.3*. Therefore, *QTSS.sicau-2D.1* and *QTSS.sicau-2D.3* are probably novel QTL that can be used for further fine mapping and genetic analysis.

In wheat, the development of polymorphism markers based on QTL is an effective method for molecular-assisted breeding (Roussel *et al.*, 2005); so, the three QTL for TSS identified in this study may be used for the breeding of high yield wheat varieties. Furthermore, the results revealed that SHW-L1 contributed positively to all the three major loci. Hence, future breeding programs can use the QTL-associated markers to fully exploit the genetic potential of QTL in increasing SHW-L1production.

A total of 23 *A. thaliana* gene homologs were predicted in the three QTL intervals. The results of GO annotation suggest that seven candidate genes deserve our attention; these are: *LECRK42*, *AT2G34930*, *PME21*, *COBL7*, *PIP5K4* (located in the *QTSS.sicau-2D.2* intervals), *CRK8* (located in the *QTSS.sicau-2D.3* intervals), and *RPPL1* (located in the *QTSS.sicau-2D.1* intervals). Among them, *LECRK42*, *PME21*, and *PIP5K4* play critical roles in pollen and pollen tube development (Sousa *et al.*, 2008; Wan *et al.*, 2008; Oo *et al.*, 2014); *AT2G34930* encodes cell wall proteins in the apoplastic fluids of rosettes (Boudart *et al.*, 2005); and *COBL7* influences the development and function of the gynoecium (Scutt *et al.*, 2003). Pollen and flower development is closely related to flowering time, and flowering time genes affect ear differentiation, including TSS (Jiang *et al.*, 1982). Moreover, differentiation of TSS indicates a switch from vegetative to reproductive growth (Li, 1976). Interestingly, *CRK8* is involved in reproductive signal transduction (Zhao *et al.*, 2011), and *RPPL1*, which interacts with *GRF2*, plays crucial roles in controlling growth and development in plants (Gökirmak *et al.*, 2015; Ghorbel *et al.*, 2017). For all these reasons, the seven candidate genes located in the three QTL intervals were considered to be closely related to TSS, which validates the accuracy of our results, provides reference for future map-based cloning experiments, and helps to better understand the genetic mechanism of spikelet growth and development in wheat.

## Conclusions

In this study, we provided a strategy of identifying QTL by combining new molecular data with phenotypic data, and identified two novel QTL for TSS. A total of seven candidate genes associated with TSS were predicted. Overall, our data provides insight into the genetic basis of TSS, which might accelerate the development of high-yielding wheat cultivars.

## Supplementary material

The following online material is available for this article

Table S1 SNPs and candidate genes significantly associated with Total spikelet number per spike (TSS) QTL.

*Associate Editor: Everaldo Gonçalves de Barros*
