# Supplementary material for: Mapping of QTL for total spikelet number per spike on chromosome 2D in wheat using a high-density genetic map
Source: Genet Mol Biol. 2019 Nov 14;42(3):603–10. doi: 10.1590/1678-4685-GMB-2018-0122 (PMC6905451; doi:10.1590/1678-4685-GMB-2018-0122)
Supplement: Supplementary file 1 [file 1415-4757-GMB-42-3-2018-0122-suppl1.pdf]

Supplementary Material to "Mapping of QTL for total spikelet number per spike on chromosome 2D in wheat using a high-density genetic map"

Table S1 - SNPs and candidate genes significantly associated with Total spikelet number per spike (TSS) QTL.

QTL	Marker interval	Chr_arm_ENSEMBL[a]	Locus (cM)[b]	Candidate genes name[c]	Annonation	Gene ID	Gene Ontology(GO)[d]			KEGG Pathway[e]
							Biological process	Molecular function	Cellular component	
QTSS.sicau-2D.1	AX94814133-AX110571866	2DS	10.45-11.72	TRIAE_CS42_2DS_TGACv1_177910_AA0587020	Arabidopsis thaliana (thale cress) gene RPPL1	AT3G14470	\	[GO:0043531]ADP binding	\	\
				TRIAE_CS42_2DS_TGACv1_177910_AA0586990	Arabidopsis thaliana (thale cress) gene CKR6	AT4G38830	GO:0042742defense response to bacterium	\	\	\
QTSS.sicau-2D.2	gpw4473-wPt740855	2DS	32.64-33.67	TRIAE_CS42_2DS_TGACv1_177373_AA0574990	Arabidopsis thaliana (thale cress) gene LECRK42	AT3G53810	[GO:0009555]pollen development	\	\	\
				TRIAE_CS42_2DS_TGACv1_177544_AA0579700	Arabidopsis thaliana (thale cress) gene AtRLP7	AT1G47890	\	[GO:0016301]kinase activity	\	\
				TRIAE_CS42_2DS_TGACv1_179152_AA0604760	Arabidopsis thaliana (thale cress) gene SDR2A	AT3G51680	\	[GO:0016491]oxidoreductase activity	\	\
				TRIAE_CS42_2DS_TGACv1_177581_AA0580490	Arabidopsis thaliana (thale cress) gene AT2G13980	AT2G13980	\	[GO:0003676]nucleic acid binding	\	\

QTL	Marker interval	Chr_arm_ENSEMBL[a]	Locus (cM)[b]	Candidate genes name[c]	Annonation	Gene ID	Gene Ontology(GO)[d]			KEGG Pathway[e]
							Biological process	Molecular function	Cellular component	
				orf173	Arabidopsis thaliana (thale cress) gene AT2G07689	AT2G07689	[GO:0006120]mitochondrial electron transport, NADH to ubiquinone	\	\	\
				atp1	Arabidopsis thaliana (thale cress) gene ATP1	ATMG01190	\	\	\	[ath00190] Oxidative phosphorylation
				TRIAE_CS42_2DS_TGACv1_178525_AA0597050	Arabidopsis thaliana (thale cress) gene PME53	AT5G19730	\	[GO:0045330]aspartyl esterase activity	\	[ath00040] Pentose and glucuronate interconversions
				TRIAE_CS42_2DS_TGACv1_178525_AA0597060	Arabidopsis thaliana (thale cress) gene BDG3	AT4G24140	\	[GO:0016787]hydrolase activity	\	\
				TRIAE_CS42_2DS_TGACv1_178369_AA0594670	Arabidopsis thaliana (thale cress) gene AT5G33406	AT5G33406	\	[GO:0046983]protein dimerization activity	\	\
				TRIAE_CS42_2DS_TGACv1_178820_AA0601350	Arabidopsis thaliana (thale cress) gene SCPL40	AT3G63470	\	[GO:0004185]serine-type carboxypeptidase activity	\	\
				TRIAE_CS42_2DS_TGACv1_177420_AA0576610	Arabidopsis thaliana (thale cress) gene TMKL1	AT3G24660	[GO:0007169]transmembrane receptor protein tyrosine kinase signaling pathway	\	\	\
				TRIAE_CS42_2DS_TGACv1_177420_AA0576620	Arabidopsis thaliana (thale cress)	AT4G29780	\	\	\	\

QTL	Marker interval	Chr_arm_ENSEMBL[a]	Locus (cM)[b]	Candidate genes name[c]	Annonation	Gene ID	Gene Ontology(GO)[d]			KEGG Pathway[e]
							Biological process	Molecular function	Cellular component	
					gene AT4G29780					
				TRIAE_CS42_2DS_TGACv1_177420_AA0576640	Arabidopsis thaliana (thale cress) gene AT5G01740	AT5G01740	\	\	\	\
				TRIAE_CS42_2DS_TGACv1_177268_AA0571460	Arabidopsis thaliana (thale cress) gene AT3G08840	AT3G08840	\	\	\	\
				TRIAE_CS42_2DS_TGACv1_177172_AA0567770	Arabidopsis thaliana (thale cress) gene AT1G68580	AT1G68580	\	[GO:0003682]chromatin binding	\	\
				TRIAE_CS42_2DS_TGACv1_177172_AA0567780	Arabidopsis thaliana (thale cress) gene AT2G34930	AT2G34930	[GO:0050832]defense response to fungus	\	\	\
				TRIAE_CS42_2DS_TGACv1_177172_AA0567810	Arabidopsis thaliana (thale cress) gene ACR2	AT5G25320	\	[GO:0016597]amino acid binding	\	\
				TRIAE_CS42_2DS_TGACv1_177172_AA0567820	Arabidopsis thaliana (thale cress) gene PME21	AT3G05610	\	[GO:0045330]aspartyl esterase activity	\	[ath00040] Pentose and glucuronate interconversions
				TRIAE_CS42_2DS_TGACv1_180706_AA0611240	Arabidopsis thaliana	AT4G16120	[GO:0010215]cellulose microfibril organization	\	\	\

QTL	Marker interval	Chr_arm_ENSEMBL[a]	Locus (cM)[b]	Candidate genes name[c]	Annonation	Gene ID	Gene Ontology(GO)[d]			KEGG Pathway[e]
							Biological process	Molecular function	Cellular component	
					(thale cress) gene COBL7					
QTSS.sicau-2D.3	AX110089401-AX94499721	2DS	53.70-53.99	TRIAE_CS42_2DS_TGACv1_178226_AA0592980	Arabidopsis thaliana (thale cress) gene PIP5K4	AT3G56960	\	[GO:0016308]1-phosphatidylinositol-4-phosphate 5-kinase activity	\	\
				TRIAE_CS42_2DS_TGACv1_179035_AA0603780	Arabidopsis thaliana (thale cress) gene CRK8	AT4G23160	\	\	\	\

a, chromosome location in hexaploid wheat from Blast program of Ensemblplant
b, genetic distance in RIL
c, identified genes in the QTL interval from Blast program of Ensemblplant
d, the best blast GO Annotate results from Annotate program of KOBAS 3.0
e, the best blast KEGG Pathway Identify results from Identify program of KOBAS 3.0
